# Effect of vermicompost and lime on faba bean (*Vicia faba* L.) grain yield and soil properties on non-responsive acidic soils of Western Amhara, Ethiopia

**DOI:** 10.1371/journal.pone.0334687

**Published:** 2025-10-21

**Authors:** Beamlaku Alemayehu, Zerfu Bazie, Tadele Amare, Erkihun Alemu, Abere Tenagne, Tarekegn Yibabie, Zelalem Addis, Anteneh Abewa, Abreham Awoke

**Affiliations:** Adet Agricultural Research Center, Adet, Ethiopia; Nepal Agricultural Research Council, NEPAL

## Abstract

Soil acidity is a global problem that limits crop production worldwide. It is the major crop yield-limiting factor in Ethiopia. The experiment was conducted in the Guagusa Shikudad district in western Amhara during the 2021 and 2022 cropping seasons to improve the productivity of faba bean through integrated vermicompost and lime applications. The spacing between rows and plants was 40 and 10 cm, respectively and the gross plot size was 8.4 m². The treatments were zero, half and full lime factorially combined with 0, 5, 10, and 15 t ha ⁻ ¹ vermicompost. Vermicompost and lime were applied separately in rows at planting. The experiment was laid out in a randomized complete block design with three replications. Before planting, a composite surface soil sample at 0–20 cm depth and after harvest from each plot was collected for the determination of soil chemical properties. The soil analysis result indicated that vermicompost and lime significantly increased soil pH and decreased exchangeable acidity. The result also revealed vermicompost and lime significantly (p < 0.001) increased faba bean grain and biomass yield. The maximum faba bean grain yield (2.41 t ha ⁻ ¹) was recorded from the applied 10 t ha ⁻ ¹ vermicompost and full dose of lime (5.6 t ha ⁻ ¹), while the maximum faba bean biomass (5.90 t ha ⁻ ¹) was recorded from the treatment of 15 t ha ⁻ ¹ vermicompost and full dose of lime applied. The minimum grain and biomass yield of faba bean was recorded from the control (vermicompost and lime not applied). Application of 5 t ha ⁻ ¹ vermicompost and a full dose of lime gave an optimum and economical faba bean grain yield. Application of integrated organic and inorganic fertilizers with lime is suggested for the improvement of faba bean grain yield by restoring non-responsive, strongly acidic agricultural soils in the study area and similar agroecology.

## 1. Introduction

Soil acidity is a global problem that limits crop production worldwide. Approximately 30–40% of the world’s total arable land and more than 70% of potential arable land are affected by soil acidity [1]. Soil acidity is also the major crop yield-limiting factor in the Ethiopian highlands. About 43% of Ethiopia’s agricultural land is affected by soil acidity [2,3]. From this, about 28% of the country’s potential arable land is affected by strong soil acidity with a pH of < 5.5, whereas the remaining 15% falls into strongly to moderately acidic soil with a pH of 5.5–6.7 [2,3]. In acidic soils, crop production is restricted by a combination of nutrient deficiencies and mineral toxicity. Acidic soils are deficient in essential nutrients, primarily phosphorus, and lack basic cations such as calcium, magnesium, and potassium [4]. On acidic soils, crops can be stunted and not very responsive to fertilizers, resulting in low yields.

Faba bean is the most important highland pulse crop in Ethiopia. It is an economically high-value crop and its seed serves as a protein source in the cereal-based Ethiopian diet [5]. It also has a great contribution to sustainable soil fertility management due to its ability to fix atmospheric N₂ to be available to plants. Ethiopia is the second-largest faba bean-producing country next to China [5]. However, its average yield is very low compared to other developed countries. This is due to soil acidity and poor soil fertility (low organic matter) problems in high-rainfall areas. Faba bean best grow in a desirable pH of 5.5–6.7 (slightly acidic to moderately acidic). These are the critical problems in Ethiopia in general and Western Amhara in particular.

Lime application potentially improved soil acidity and crop yields. It is the most widely used practice globally to neutralize excessive hydrogen ions (H^+^) in the soil solution and enhance crop productivity [6]. Adet Agricultural Research Center (Adet, Ethiopia) was also successful in improving wheat production by applying one-fourth (¼) of the lime from the full dose in micro-dosing (row) at planting (based on the exchangeable acidity method) [7]. However, the endeavor to pre-scale up using ¼ of the lime application for faba bean production in acidic soils of the Machakel and Gozamen districts of the East Gojam Zone, Amhara region, was not successful. Following this work, the low productivity of faba beans has been observed in the strongly acidic soils with low organic matter content in the highlands of Gojam, Ethiopia. Both soil acidity and low soil fertility, including insufficient organic matter, are major yield-limiting factors in soils of this area.

The major causes of soil acidity and low soil fertility are soil erosion and the continuous removal of crop residues in agricultural soils [8]. Therefore, enhancing crop yield by improving soil acidity through lime amendments and the application of organic and inorganic fertilizers has been recommended in degraded soils [9,10]. However, the information on soil acidity amendment using integrated application of vermicompost and lime is insufficient in Ethiopia, particularly in the strongly acidic soils of western Amhara. Even though soil amendment using lime was tested before this experiment and the results showed that applying lime only did not improve the growth and yield of faba beans in strongly acidic soils. Hereafter, this research was designed and conducted to test the hypothesis that integrated vermicompost and lime applications would improve soil acidity and faba bean grain yield in strongly acidic soils. Therefore, on-farm experiment was planned to determine the appropriate combined vermicompost and lime rates to improve the productivity of faba bean in acidic soils through integrated vermicompost and lime applications.

## 2. Materials and methods

### 2.1. Description of the study area

The experiment was conducted on four sites in the Guagusa Shikudad district for two cropping seasons (2021–2022). According to the World Reference Base for Soil Resources (WRB) classification, the soil type of the study area is Nitisols with the Phaeozem reference soil group [11]. Nitisols, characterized by their deep, reddish, well-structured, and clayey nature, are often found in tropical and subtropical regions with good drainage. Phaeozems are characterized by dark-colored t opsoil [11]. The Guagusa Shikudad district was found in the Awi zone of the Amhara National Regional State. Geographically, the site is located between 37º 1’ 44“ and 37º 2’ 29” East longitude and 11º 32’ 54” North latitude. The altitude ranges from 2482 to 2800 m above sea level.

### 2.2. Climate

**Fig 1:**
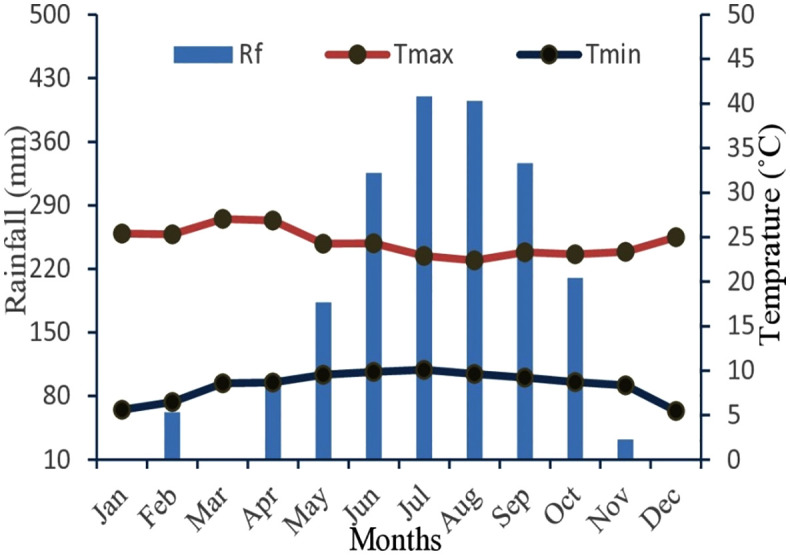
Climate data of the study area during the experimentation Ethiopian Meteorological Service Agency (EMSA), Northwest branch, Bahir Dar, 2021.

The study sites are located in the Dega agro-climatic zone, characterized by a dry season that extends from October to May in the mid-highlands. The rainy season, which is called Kiremet (summer), starts in June and ends in September. The total rainfall was 2100 mm during the experimental year, in which the maximum (410 mm) was recorded in July, followed by 405 mm in August [12]. The mean daily maximum and minimum temperature ranges from 22.4°C in August to 27°C in March and from 5.5°C in December to 10.1°C in July, respectively [12]. In general, the mean annual minimum and maximum temperatures were 8°C and 24°C, respectively (Fig 1).

### 2.3. Experimental design and materials used

The experiment was arranged in a factorial randomized complete block design with three replications. The *Wolkie* variety was used as a test crop. The gross plot size was 2.8 m width x 3 m length with 40 and 10 cm spacing between rows and plants, respectively. During the experimentation, 20 kg ha^-1^ nitrogen (starter) and 46 kg ha^-1^ P_2_O_5_ nutrients were applied for all plots. Vermicompost and lime were applied separately in rows at planting for all experimental sites.

### 2.4. Treatment setup

In the experiment, 3 levels of lime (zero, half, and full) were factorially combined with 4 levels of vermicompost (0, 5, 10, and 15 t ha ⁻ ¹). The amount of lime required per hectare (full dose) was calculated based on the modified model of [13], as indicated in [14], as shown in equation 1. Based on this calculation, the full dose of lime applied during the experiment was 5.6 t ha ⁻ ¹.


$$\text{LR},\;\text{Ca}{\text{CO}}_{\text{3}}\;(\text{kg}\;\text{ha}{-}^{\text{1}})\;=\;\frac{\left(EA*\text{0}.\text{2}*BD(Mg/m\text{3})*\text{1}\text{0}\text{0}\text{0}\text{0}\right)*\text{1}\text{0}\text{0}\text{0}}{\text{2}\text{0}\text{0}\text{0}}*\text{1}.\text{5}$$ (1)

Where LR = Lime Requirement, EA = Exchangeable acidity (cmol (^+^) kg ⁻ ¹ of soil), 0.2 = root depth (m) or lime incorporation depth, BD = Bulk density (g cm ⁻ ³), 10000 = Area in a hectare, 1000 = conversion factor to convert bulk density from g cm ⁻ ³ to mega gram m ⁻ ³ and 2000 = conversion factor to convert exchangeable acidity from per kilogram of soil to per hectare.

### 2.5. Nutrient content analysis of vermicompost

The materials used for vermicompost were chopped banana stems and leaves, tomato stems, leaves and fruit, dry grass, and cow dung. Before vermicompost application, the nutrient content analysis was done (Table 1).

**Table 1:** Nutrient contents of vermicompost used in the experiment.

Parameters	Values
pH (H_2_O)	7.6
TN (%)	1.3
CEC (c mol^ + ^kg^-1^ soil)	65.7
OC (%)	18.2
Ava. P (mg kg^-1^)	477.6
C: N	14:1

Where, pH = power of hydrogen, C: N = carbon to nitrogen ratio, Ava. P = Available phosphorus, TN = Total nitrogen, OC = Organic carbon, CEC = Cation exchange capacity.

### 2.6. Soil and agronomic data collections

#### 2.6.1. Soil sampling and analysis.

Before planting, a composite surface soil sample at 0–20 cm depth and after harvest from each plots were collected for the determination of soil chemical properties. The collected soil samples were air-dried and crushed to pass through a 2-mm sieve. In this study, selected soil parameters, including soil pH-H_2_O, exchangeable acidity, available phosphorus (Ava. P in mg kg ⁻ ¹), total nitrogen (TN in %), organic carbon (OC in %) and cation exchange capacity (CEC in cmol+ kg ⁻ ¹ soil), were analyzed at the Adet Agricultural Research Center’s soil laboratory. Soil reaction (pH H₂O) was measured in 1:2.5 soil-to-water suspensions following the procedure used by Sertsu and Bekele [15]. Soil organic carbon (OC) content was measured by the wet digestion method using the Walkley and Black method [16]. The total nitrogen was estimated using the Kjeldahl method [17], while the available phosphorus was analyzed by following the Olsen method [18]. The ammonium acetate extraction procedures were used to determine the soil cation exchange capacity [19].

#### 2.6.2. Agronomic data collections.

Total aboveground biomass and grain yield were measured for the faba bean crop. The planting date of faba bean was July 2 – July 6 and the harvesting date was December 5 – December 9 for both cropping seasons (2021 and 2022). Harvesting was done from the middle five rows, with the outside rows left as buffers to prevent border effects. Plants harvested from the net plot area were sun-dried to a constant weight and converted to kg per hectare for statistical analysis. The grain yield was also calculated after threshing the biomass harvested from the net plot area and converted into kilograms per hectare. We adjusted the grain yield to 10% moisture.

### 2.7. Partial budget analysis

A financial return analysis was performed to investigate the economic feasibility of liming for faba bean production. The partial budget analysis was calculated based on CIMMYT economics training manual [20]. The average grain and straw yields of faba bean were adjusted downwards by 10%, as available evidence showed that the grain yields obtained from farmers’ fields under their own management could be reduced by 10% compared to grain yields from experimental plots from the same treatment. The output data (grain yield and straw yield) at threshing time and input data (market price for labor and lime) during the experimental year were collected for two consecutive seasons (2021 and 2022) and averaged for analysis. The total variable costs were calculated by adding the costs of lime, labor and vermicompost. Dominance analysis was conducted to identify the most cost-effective options. To perform this, the treatments were arranged in ascending order by total variable costs. The net benefit for each treatment was calculated and the total variable costs of each treatment were subtracted from each gross net benefit. When the net benefit of one treatment is less than the net benefit of a treatment with lower total variable costs, the treatment with higher total variable costs is rejected, and the treatment is considered dominant. The minimum acceptable marginal rate of return (MRR) is over 50% to 100% for a treatment to be observed as an economically feasible option for smallholder farmers [20]. The partial budget analysis of this experiment is presented in (Table 2).

**Table 2:** Partial budget analysis.

Lime rates t ha^-1^	Vermicompost rates t ha^-1^	Gross income (ETB ha^-1^)	TVC (ETB ha^-1^)	Net benefit (ETB ha^-1^)	Dominance analysis	MRR (%)	Rank
Zero (0)	0	22399	0	22399			
Half (2.8)	0	52139	14200	37939		109	2
Full (5.6)	0	54518	28400	26118	D		
Zero (0)	5	95508	10400	85108	D		
Half (2.8)	5	107780	24600	83180	D		
Full (5.6)	5	141858	38800	103058		140	1
Zero (0)	10	149512	20800	128712	D		
Half (2.8)	10	153450	35000	118450	D		
Full (5.6)	10	166594	49400	117194	D		
Zero (0)	15	151978	31200	120778	D		
Half (2.8)	15	166410	45400	121010			
Full (5.6)	15	177278	59600	117678	D		

*Dominated; * 1USD = 52 Ethiopian birr. The price of Faba bean grain was 48birr kg^−1^; the price of Faba bean straw was 20birr kg^−1^. The market price of lime was 4.7birr kg^−1^; the price of vermicompost was 2 birr kg^−1^ the Labor cost for spreading lime was 100 ETB per person-day at the time of the study.Where, ETB ha^−1^ Ethiopian birr per hectare, TVC = Total variable cost, MRR = Marginal rate of return.

### 2.8. Statistical data analysis

After homogeneity and normality tests were done, the essential agronomic data collected from field experiments for each parameter were subjected to analysis of variance (ANOVA) using R programming software. Treatment means were separated based on the least significant difference (LSD) test at P ≤ 0.05.

## 3. Results

### 3.1. Effects of lime and vermicompost on soil chemical properties

**Fig 2:**
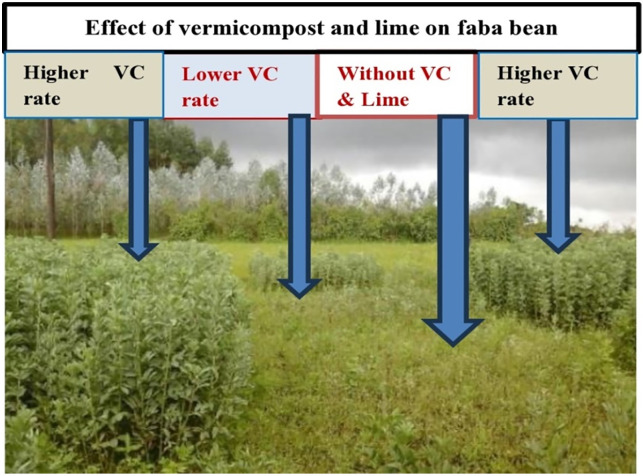
Vegetative response of Faba bean to the applied vermicompost and lime in non-responsive strongly acidic soils at Guagusa Shikudad district.

The vegetative response of faba bean to the applied vermicompost and lime during experimentation showed that without organic matter and lime application, faba bean cannot produce a grain yield in non-responsive strongly acidic soils (Fig 2). The soil analysis result (after harvest) showed that application of vermicompost and lime didn’t bring a significant change to soil-available phosphorus, total nitrogen, organic carbon, and cation exchange capacity in the experimental sites (Table 3). This might be due to the short period of the experiment to observe the residual effect of vermicompost on those soil properties. The maximum faba bean grain (2.41 t ha ⁻ ¹) was recorded from the treatment that had 10 t ha ⁻ ¹ of vermicompost and half a dose of lime (2.8 t ha ⁻ ¹) applied. Whereas the maximum faba bean biomass (5.90 t ha ⁻ ¹) was recorded from 15 t ha ⁻ ¹ of vermicompost and half a dose of lime applied. On the other hand, the minimum grain and biomass yield was recorded from the control (without vermicompost and lime) (Table 4).

**Table 3:** Selected soil chemical properties after harvest.

Site 1										
Soil parameters		Vermicompost rates t ha^-1^				Lime rates t ha^-1^			Ratings	References
	Initial	0	5	10	15	Zero	Half	Full		
Ava. P	10	8	6	7	8	7	8	7	Low	[35]
TN	0.2	0.2	0.2	0.2	0.2	0.2	0.2	0.2	Low	[36]
OC	1.6	2.1	2.1	2	1.9	2	2	2.1	Low	[37]
CEC	33	36	36	36	36	36	36	36	High	[36]
Site 2										
Ava. P	10	9	10	9	8	9	9	9	Low	[35]
TN	0.2	0.2	0.2	0.2	0.2	0.2	0.2	0.2	Low	[36]
OC	3.5	2.1	2.2	2.1	1.9	2.1	2	2.2	Low	[37]
CEC	36	33	35	34	34	34	34	35	High	[36]

Ava. P = Available phosphorus (mg kg^-1^), TN = Total nitrogen (%), OC = Organic carbon (%), CEC = Cation exchange capacity (c mol^+^ kg^-1^ soil).

**Table 4:** Main effects of vermicompost and lime on faba bean grain and biomass yield (t ha^-1^).

Treatments	2021 combined		2022 combined		Combined over the years	
Vermicompost rates (t ha^-1^)	GY	BY	GY	BY	GY	BY
0	0.17c	0.24c	1.00b	2.66c	0.58c	1.45c
5	1.31b	2.45b	1.92a	5.36b	1.61b	3.90b
10	2.42a	3.93a	2.40a	6.65a	2.41a	5.29a
15	2.22a	4.46a	2.37a	7.33a	2.29a	5.90a
Pr.	***	***	***	***	***	***
Lime rates (t ha^-1^) based on Ex. Acidity						
Zero	1.25c	2.31b	1.64b	5.14a	1.45b	3.72b
Half (2.8)	1.51b	2.55b	2.09a	5.56a	1.80a	4.05ab
Full (5.6)	1.82a	3.46a	2.03ab	5.80a	1.93a	4.63a
Pr.	***	NS	NS	NS	***	NS
VC*L	*	NS	NS	NS	NS	NS
CV (%)	27.8	29.8	37.1	30.9	37.9	47.3

Where VC = Vermicompost, L = Lime, GY = Grain yield, BY = Biomass yield, Ex. Acidity = Exchangeable acidity, CV = Coefficient of Variation, * = significant at 0.05, ** = significant at 0.01, *** = significant at < 0.01.

Where VC = vermicompost

### 3.2. Effects of lime and vermicompost on soil acidity

**Fig 3:**
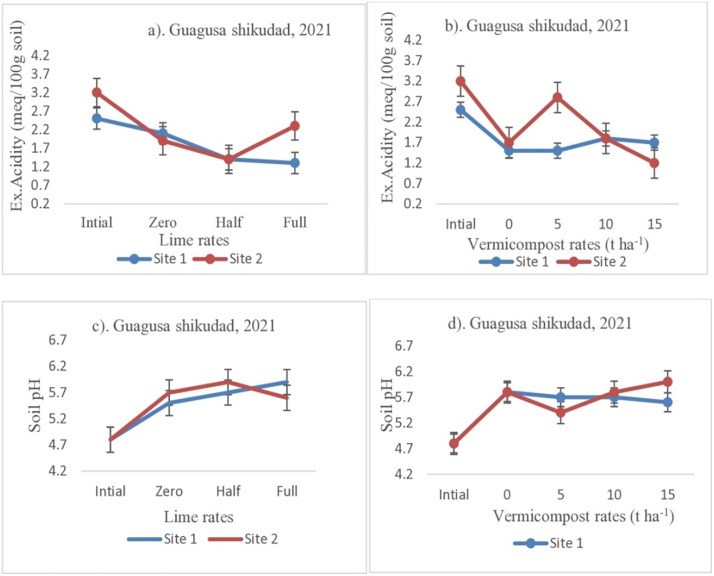
Effect of Lime and Vermicompost on Soil pH and Ex. Acidity at Guagusha Shikudad district.

**Fig 4:**
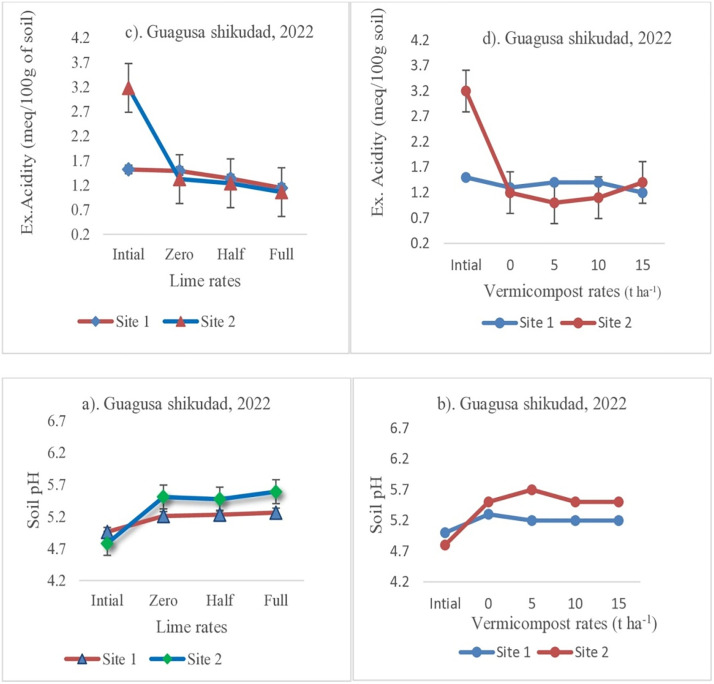
Effect of lime and vermicompost on soil pH and Ex. Acidity in Guagusa Shikudad district.

The soil analysis result showed that the application of vermicompost and lime had a significant effect on soil pH and exchangeable acidity (Figs 3 and 4). Both amendments decreased exchangeable acidity, thereby increasing soil pH (Figs 3 and 4). A rise in soil pH due to vermicompost and lime applications could be due to basic cations released from mineralization of vermicompost and lime, replacing acidic cations on soil colloids. The soil results also showed that as the lime rate increases, the exchangeable acidity decreases by 33% at site 1 and 32% at site 2 (Fig 3a) and increases the soil pH by 3.6% at site 1 and site 2 (Fig 3c) in the first year. Likewise, as the lime rate increased, the exchangeable acidity decreased by 27% at site 1 and 15% at site 2 (Fig 4c) and increased soil pH by 1.8% at site 1 and site 2 (Fig 4a) in the second year. Vermicompost application alone decreased soil exchangeable acidity by 41% at site 2 and 16% at site 1 (Fig 3a) and by 59% at site 2 and 13% at site 1 (Fig 4c) in 2021 and 2022, respectively. Similarly, lime application alone decreased soil exchangeable acidity by 32% at site 1 and 47% at site 2 (Fig 3a). On the other hand, the soil pH increased by 17% at both sites 1 and 2 (Fig 3d) in 2021. An increase in soil pH by 8% at site 1 and 15% at site 2 (Fig 4a) and also decreases in soil exchangeable acidity by 62% at site 2 and 52% at site 1 (Fig 4c) were observed in the plots that received a half dose of lime applied in 2022.

The combined soil analysis result showed that soil pH was raised and the exchangeable acidity was decreased significantly when lime and vermicompost were applied (Table 5). These results revealed that applying half and full lime raised the soil pH by 5.4% and 8.6%, respectively, and significantly decreased soil exchangeable acidity by 50.0% and 63.6%, respectively, compared to the treatment where vermicompost and lime were not applied. The application of 15 t ha ⁻ ¹ of vermicompost raised soil pH by 7.0% and reduced exchangeable acidity by 54.6% compared to the control treatment (without vermicompost and lime) (Table 5).

**Table 5:** The combined effect of vermicompost and lime on soil pH and exchangeable acidity.

Treatments	pH	Exc.Ac
VC0LF	5.8	0.8
VC0LH	5.6	1.1
VC0LZ	5.3	2.2
VC1LF	5.8	0.9
VC1LH	5.4	1.4
VC1LZ	5.4	1.9
VC2LF	5.6	1.1
VC2LH	5.7	0.9
VC2LZ	5.3	2.1
VC3LF	5.7	1.0
VC3LH	5.5	1.2
VC3LZ	5.4	2.1
LSD(0.05)	0.3	0.5
CV (%)	6.2	49.1
pr.	**	**
Std Error (±)	0.1	0.3

Where, VC0 = Vermicompost zero, VC1 = Vermicompost 5 t ha^-1^, VC2 = Vermicompost 10 t ha^-1^, VC3 = Vermicompost 15 t ha^-1^, LZ = Lime zero, LH = Lime half, LF = Lime full, pH = power of hydrogen (soil reaction), Exc.Ac = exchangeableacidity(cmol(+)kg^-1^*).*

### 3.3. Response of faba bean grain and biomass yield to the applied lime and vermicompost

**Fig 5:**
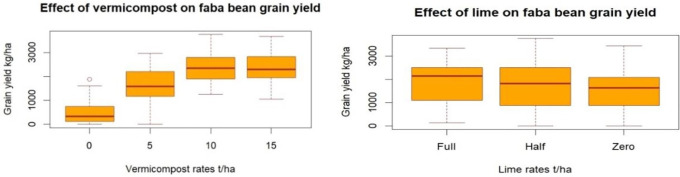
Effect of vermicompost and lime on Faba bean grain yield.

The results revealed that faba bean grain and biomass yield significantly (p < 0.001) improved through the applied vermicompost and lime (Fig 5 and Table 4). However, the combined analysis showed that vermicompost and lime had no interaction effect (Table 4). This may be due to the nutrients supplied by vermicompost meeting the crop’s requirements; under such conditions, lime may not have a significant effect on the nutrient dynamics. These results also showed that faba bean grain and biomass yield did not show a statistically significant difference between the applied half and full dose of lime (Fig 5 and Table 4). This result also showed that application of 10 and 15 t ha ⁻ ¹ has no statistically significant difference on faba bean grain and biomass yield (Table 4). The combined CV of faba bean grain and biomass was shown to be high; this is due to the presence of high grain and biomass yield variability in the cropping seasons, especially in 2021, when grain yield was not recorded at plots where VC was not applied (Fig 5 and Table 4).

The ANOVA result showed that application of 10 and 15 t ha ⁻ ¹ of vermicompost didn’t have a statistically significant difference on faba bean grain and biomass yield (Fig 5 and Table 3). Maximum and significant grain yield (2.41 t ha^-1^) was obtained by applying 10 t ha^-1^ of vermicompost. In contrast, a yield of 0.58 t ha ⁻ ¹ was recorded without vermicompost, despite the addition of lime. A higher and significant grain yield (1.93 t ha^-1^) was observed with the full lime rate amendment of 5.6 t ha^-1^. Additionally, a grain yield of 1.45 t ha ⁻ ¹ was achieved without lime amendment, which was solely due to the application of vermicompost. However, this yield was recorded from vermicompost applied only. The partial budget analysis indicated that the application of 5 t ha^-1^ vermicompost with a full dose of lime (5.6 t ha^-1^) showed the most economical optimum rate, which gave a net benefit of 103, 058 ETB ha^-1^ and a higher marginal rate of return, which is 140% (Table 4).

## 4. Discussion

### 4.1. Contribution of lime and vermicompost on soil health

Soil pH and exchangeable acidity are key indicators of soil acidity in agricultural soils. This study articulates the role of organic and lime amendments on soil health and crop productivity in non-responsive acidic soils of the Ethiopian highlands. The application of both lime and vermicompost significantly improves soil health, particularly by enhancing soil pH and reducing exchangeable acidity, which in turn positively affects faba bean grain yield in the study area. These results are in line with the findings of Alemu *et al*. [21], who reported that the application of 2.2 t ha^-1^ lime raises the soil pH by 4% in acidic agricultural soils of Ethiopia. Regasa *et al.* [22] also reported similar results that the combined application of lime and vermicompost improved soil pH by 27% and reduced exchangeable acidity by 70%.

These results are consistent with the findings of Sosena and Sheleme [23], who reported that lime application significantly reduced soil exchangeable acidity and increased soil pH. The results also agree with the findings of Alemu *et al*. [21], who reported that the application of lime significantly decreases soil exchangeable acidity in non-responsive soils. These results are also consistent with [24,25], who described vermicompost and lime application improved soil acidity by reducing exchangeable acidity and raising the soil pH. In the same way, Terefe *et al*. [26] also reported similar results, who reported that combined vermicompost and lime applications increased soil pH by 12.1% and decreased exchangeable acidity by 92.8%. These results also agreed with the findings of Abebe *et al*. [27], who reported that the integrated application of 4 t ha^-1^ lime and 5 t ha^-1^ vermicompost significantly reduced soil exchangeable acidity and increased soil pH.

Similarly, Ejigu *et al*. [28] concluded that the application of organic fertilizer significantly improved soil pH and exchangeable acidity in strongly acidic soils. These results are also in line with the findings of Wabela *et al*. [29], who reported that the combined application of vermicompost with inorganic fertilizer improved soil pH as compared to the sole fertilizer applied. A rise in pH could be related to the release of basic cations like Ca^+2^, Mg^+2^, K^+^ and Na^+^ from vermicompost mineralization as well as lime addition, which can replace acidic ions (H^+^, Al^+3^ and Fe^+3^) on the surface of soil colloids [28]. In this study, the results showed that vermicomposting is a very important yield-limiting factor, followed by lime amendment for soil health improvement. Combining organic and inorganic fertilizers with lime is an important strategy for enhancing soil health and increasing crop yields. Research has shown that integrated soil fertility management (ISFM) can greatly boost crop productivity and soil health in the Ethiopian Highlands [30].

### 4.2. Role of lime and vermicompost amendment on faba bean productivity

Performance and grain yield of faba bean significantly increased through integrated nutrient application or addition of lime, vermicompost and chemical fertilizers compared to the control treatment. The addition of vermicompost, chemical fertilizer with lime amendment significantly enhances faba bean productivity. Application of 10 t ha^-1^ vermicompost increased faba bean grain yield by 75.6% as compared to the treatment where vermicompost was not applied. On the other hand, 5.6 t ha^-1^ (full dose) lime application increased faba bean grain yield by 24.9% as compared to the treatment where lime was not applied (Table 3). These results agreed with the findings of Bekele *et al*. [24], who reported that the combined use of vermicompost and lime increased maize grain yield almost double compared to the treatment where vermicompost and lime were not applied. These results are also in agreement with the findings of Ejigu *et al*. [28], who reported that combined applications of compost and mineral fertilizers increased yield and yield components of maize as compared to sole fertilizer applied.

These results are also in line with the findings of Geleta and Bekele [31], who reported that the application of 4 t ha^-1^ lime in combination with 120 kg ha^-1^ nitrogen, phosphorus, sulfur and boron containing fertilizer gave maximum faba bean grain yield. Fekadu *et al*. [32] also reported that 4 t ha^-1^ farmyard manure, 15 kg P ha^-1^ and 3.6 t ha^-1^ lime applications gave 47% grain yield advantage as compared to the control treatment. Similarly, Habtamu *et al*. [33] also reported that half-dose vermicompost and lime applications significantly improved N and P nutrient uptake and faba bean grain yield. Wabela *et al*. [29] also reported similar results that applications of integrated organic and mineral fertilizers increased common bean grain yield as compared to sole fertilizer applied. The increased faba bean grain yield as vermicompost applied could be due to soil organic matter improved and release of higher N and P [34]. The lower grain yield from the control treatment might be due to the lower organic matter content of the soil, as the grain yield increased when vermicompost was applied.

This result indicated that faba bean production without organic matter application is very challenging in strongly acidic nonresponsive agricultural soils of the study area. This states that vermicompost followed by lime is a key production input for fababean productivity in non-responsive strongly acidic soils. The economical optimum recommendation helps the smallholder farmers to apply the minimum available organic matter and lime to produce reasonable yields in the farming system. Because there is significance shortage of organic matter as a result of competition with fuel wood sources to make meals.

## 5. Conclusions and recommendations

Vermicompost and lime applications significantly improve faba bean productivity and soil health in non-responsive acidic soils of Ethiopia. Vermicompost is a critical organic amendment for faba bean production and soil health improvement. It is also impossible to produce faba bean in non-responsive acidic soils through lime application alone under depleted soil organic matter. The lime application significantly improves soil health by amending soil exchangeable acidity and soil pH. Thus, the application of 5 t ha ⁻ ¹ of vermicompost with a full dose (5.6 t ha ⁻ ¹) of lime gave an economical optimum faba bean grain yield and soil health in the study area. Application of lime without organic matter amendment could not improve the growth and productivity of faba bean in strongly acidic soils. Therefore, it is possible to recommend the application of 5 t ha ⁻ ¹ vermicompost with 5.6 t ha ⁻ ¹ lime for economical optimum faba bean production and soil health maintenance in the study area and similar non-responsive strongly acidic soils. Hence, applying integrated soil fertility management, like organic and inorganic fertilizers with lime amendment, is suggested as a key option for optimum crop yield and soil health improvement in degraded agricultural soils of Sub-Saharan Africa and the world.

## Supporting information

S1 Data Data for faba bean. (XLSX)
